# Pain in newborn

**DOI:** 10.1186/1824-7288-40-S1-A54

**Published:** 2014-08-11

**Authors:** Lorenzo Giacchetti, Monika Stablum, Arianna De Martino, Arturo Giustardi

**Affiliations:** 1 Department of Pediatrics and Neonatology, Lugano, Switzerland; 2 Associazione Italiana La Care in Perinatologia, Italy; 3 Department of Neonatology, Mantova, Italy; 4 Department of Neonatology Merano, Italy

## 

Recent scientific studies have added more and more consistent evidence that the newborn, even if preterm, is very sensitive to the nociceptive stimulus. The baby responds with a well known physiological, metabolic and hormonal reaction, that, if repeated, may lead to short and medium term negative effects on the newborn. This vulnerability to painful stimulus, especially in preterm infants, can be explained by neurophysiological mechanisms. In spite of a well developed sensory apparatus for nociception, the descending inhibitory systems and their neurotransmitters responsible for nociceptive afferents, are deficient and immature until after the term birth. Behavioral changes and reductions in the volume of some sensitive brain areas were observed in ex preterm infants admitted to the neonatal intensive care unit; the modifications were very similar to those seen in experimental model of rat with same gestational age, exposed to early and repeated painful stimulus [1]. We consider as environmental interventions all steps that can reduce stress in the baby during a painful procedure. A variety of non pharmacologic pain-prevention and relief techniques have been shown to effectively reduce pain from minor procedures in neonates. These include use of oral sucrose/glucose, breastfeeding, non-nutritive sucking, kangaroo care, facilitated touch (holding the arms and legs in a flexed position), swaddling, and developmental care [2]. The involvement of the mother is recommended through skin-to-skin or breast-feeding during a single sampling. Several studies have shown how breastfeeding during a painful procedure reduces the stress in newborn [3].

The prevention of pain in neonates should be the goal of all caregivers, because repeated painful exposures can have deleterious consequences. Neonates at greatest risk of neurodevelopmental impairment as a result of preterm birth are also those most likely to be exposed to the greatest number of painful stimulus in the NICU. We have to improve strategies for routinely assessing pain, minimizing the number of painful procedures performed, using pharmacologic and non pharmacologic therapies for the prevention of pain associated with routine minor procedures, and eliminating pain associated with major procedures.
